# 

**Published:** 1909-03

**Authors:** 


					CONTENTS.
Page
The Long Fox Lecture: Metastatic Inflammations of the Eye.
By J. Herbert Parsons, B.S., D.Sc. (Lond.), F.R.C.S. (Eng.)
The Causes of Transient Cerebral Paralyses.
By George Parker,
M.A., M.D.Cantab.
15
The Physique of Boys.
By Bertram M. H. Rogers, M.D.,
B.Ch., B.A. Oxon.
27
Thomas Dover: Physician and Merchant Adventurer.
By J. A.Nixon,
M.B.Cantab., M.R.C.F.
31
vomiting Connected with Anaesthesia.
By A. L. Flemming, M.R.C.S.
4?
Milk from a Public Health otanapoint ? City of Bristol.
By
D. S. Davies, M.D. Lond., D.P.H.
44
Some Impurities of Vended Milks.
By I. Walker Hall, M.D. ...
The Adulteration of Milk in Bristol.
By Edward Russell, B.Sc., F.I.C.
47
The Value of Some Lactic Acid Ferments.
By Drs. Walker Hall
and W. A. Smith
56
Precautions to be Observed in Administration of Milk in Disease.
By J. Michell Clarke, MA., M.D.Cantab., r.K.C.P.
57
The Heating of Milk as it Affects the Nutrition and Health of the
Infant.
By J. M Fortescue-Brickdale, M.A., M.D.
6i
Progress of the Medical Sciences :
Surgery.
Ernest W. Hey Groves, M.B., B.Sc. Lond.
62
Laryngology and Otology.
P. Watson Williams, M.D. Lond. ...
69
Reviews of Books
7*
Editorial Notes
78
Notes on Preparations for the Sick
85
The Library of the Bristol Medico-Chirurgical Society   88
Meetings of Societies
9i
Local Medical Notes
93

				

